# Association of urinary cotinine-verified smoking status with hyperuricemia: Analysis of population-based nationally representative data

**DOI:** 10.18332/tid/127269

**Published:** 2020-10-06

**Authors:** Yunkyung Kim, Jihun Kang

**Affiliations:** 1 Department of Rheumatology, Kosin University Gospel Hospital, Kosin University, Busan, Republic of Korea; 2 Department of Family Medicine, Kosin University Gospel Hospital, Kosin University, Busan, Republic of Korea; 3 Central Institute for Medical Research, Kosin University Gospel Hospital, Busan, Republic of Korea

**Keywords:** uric acid, hyperuricemia, cotinine, smoking, KNHANES

## Abstract

**INTRODUCTION:**

Smoking status based solely on self-reporting is unreliable and might be inaccurate, particularly among women. This study investigated the association between urinary cotinine-verified smoking status and hyperuricemia in a nationwide Korean population.

**METHODS:**

This study included 5329 participants aged ≥19 years with information on smoking status, urine cotinine levels and serum uric acid. We determined smoking status according to self-reports and urinary cotinine levels. Multivariate linear regression analysis was used to measure the association between smoking exposure and serum uric acid levels. The effects of smoking on hyperuricemia were evaluated by multivariate logistic regression analysis.

**RESULTS:**

Biochemically verified active and passive smokers comprised 22% (38.7% of men and 8.8% of women) and 12.3% (11.9% of men and 12.6% of women) of the study population, respectively. While reclassification rate of active smokers was 1.4% in men, 31.8% of cotinine-verified female active smokers were self-reported never smokers. Higher uric acid levels were observed with increased tobacco exposure among women (p-trend=0.007) but not among men. After adjusting for confounders, the risk of hyperuricemia increased with tobacco exposure only in women (p-trend=0.016).

**CONCLUSIONS:**

Cotinine-verified smoking status was associated with increased serum uric acid and hyperuricemia in a dose-response manner only in women. This study might provide evidence to support the importance of smoking cessation in women with gout and further studies are necessary to elucidate the underlying mechanism of the observed association.

## INTRODUCTION

Hyperuricemia is a prerequisite condition for gout and results from decreased uric acid excretion or increased uric acid production due to increased cell turnover, high-purine diet, or deficiency of enzymes related to purine metabolism^1,2^. Growing evidence indicates that hyperuricemia is not only a pre-existing condition for gout but also a risk factor for diabetes^3^, hypertension^4^, chronic kidney disease^5^, premature death from cardiovascular disease^6^, and overall mortality^7^; thus, the increasing prevalence of hyperuricemia, especially in women, is drawing increasing attention in public health owing to its implications for chronic diseases^8^.

Serum uric acid levels are determined by the interplay of genetic and environmental factors. Several genetically-susceptible loci including solutecarrier family 2 member 9 (SLC2A9), ATP-binding cassette subfamily G member 2 (ABCG2), glucokinase regulator (GCKR) etc., are associated with uric acid excretion in kidney and individuals having risk allele of these loci showed elevated serum uric acid levels and increased risk of gout^9,10^. However, although previous studies have demonstrated the impact of various health behaviors including the consumption of heavy alcohol, high-purine diets^11^, and sugar-sweetened drinks^12^ on uric acid, the effects of smoking on serum uric acid have not been established, with conflicting results according to sex and ethnicity. The Coronary Artery Risk Development in Young Adults (CARDIA) study showed higher serum uric acid levels in female smokers than in female non-smokers^13^. Another study conducted in Japan showed that smoking was associated with increased serum uric acid levels regardless of sex^14^. However, a study using Framingham Heart Study cohort data reported that cigarette smoking had protective effects against elevated uric acid levels^15^ and a longitudinal study from Japan observed a decreased risk of hyperuricemia among smokers^16^.

Obtaining accurate data on smoking status is challenging, particularly among Asian populations because self-reported smoking status based on selfadministered questionnaires or face-to-face interviews show substantial differences in active and passive smoking rates when compared to biochemically determined smoking status, a discrepancy that is more evident among women^17,18^. While a relatively small difference between self-reported and cotinine-verified smoking rates was observed in Korean men (44.7 vs 50.0 %), this gap was larger in women (5.9 vs 13.9 %), a two-fold difference^18^. Although misclassification of smoking status should be addressed in study protocols because it could significantly impact study results, few studies have considered this issue when evaluating the effects of smoking on serum uric acid level and the risk of hyperuricemia.

In this context, the present study evaluated the association between urinary cotinine-verified active and passive smoking and hyperuricemia in a nationally representative sample of the Korean population.

## METHODS

### Study participants

The Korean National Health and Nutrition Examination Survey (KNHANES) is a nationwide cross-sectional survey of the non-institutionalized Korean population. This survey is conducted annually by the Korea Centers for Disease Control and Prevention (KCDC) to obtain information on the health and nutritional status of the Korean general population. The KNHANES consists of a health interview, a health examination, and a nutritional survey, which provide data on demographic variables, health behaviors, socioeconomic status, biochemical profiles, quality of life, and dietary intakes. The sampling plan is designed based on national census data and individuals aged ≥19 years are eligible for inclusion in the survey. A nationally representative sample was obtained with the use of a stratified multistage clustered probability sampling. The study’s protocol was approved by the institutional review board of Kosin University Gospel Hospital (IRB Number KUGH 2019-IRB 2019-10-006) and this study complied with the principles of the Declaration of Helsinki 1975. Informed consent was obtained from all study participants and all study processes were performed according to the guidelines of Strengthening the Reporting of Observational Studies in Epidemiology.

### Inclusion and exclusion criteria

We used data from the KNAHNES 2016, which included measurements of serum uric acid level for the first time. Among 10806 eligible individuals, 8150 consented to participate in the study, corresponding to a response rate of 75.4%. Inclusion criteria were participants aged ≥19 years with data on self-reported smoking status and urine cotinine and serum uric acid levels. A total of 5584 participants were identified. We excluded 255 participants according to the following criteria: a previous diagnosis of renal cell carcinoma (n=13), glomerular filtration rate of <10 mL/min/1.73 m^2^ (n=4), pregnancy (n=27), missing data on income (n=14), education (n=162), anthropometric variables such as body mass index (BMI, kg/m^2^; n=3), blood pressure (n=7), alcohol consumption (n=14), and physical activity (n=11). Finally, 5329 participants (2349 men and 2980 women) were included in the analysis.

### Data collection and measurement

Data on sociodemographic characteristics (age, sex, area of residence, household income, and education) were collected by trained research assistants during face-to-face interviews. Information regarding health behaviors (smoking status, alcohol consumption, and physical activity) was obtained from self-administered questionnaires. The areas of residence were categorized dichotomously into rural or urban areas. Household income was categorized into three groups based on a tertile distribution (low, middle, high). Education was also categorized into three groups (middle school, high school, college or higher).

We categorized smoking status as active smokers, passive smokers, and non-smokers based on selfreported smoking status and urinary cotinine levels. Although the World Health Organization (WHO) classification is widely used to categorize smoking status, biochemical markers are not taken into account in this classification, thus, the smoking status of the participants in the present study was categorized as follows: active smokers were defined as participants who reported smoking ≥100 cigarettes in their lifetime and who currently smoked; passive smokers were defined as those who did not smoke but had been exposed to tobacco smoke either in the workplace or at home during the last week; non-smokers were defined as participants who smoked <100 cigarettes during their lifetime, did not smoke currently, and had not been exposed to secondhand tobacco smoke. Because of the unreliability of self-reported smoking status, we verified each subject’s smoking status using urinary cotinine concentration. For example, participants classified as non-smokers based on the questionnaire responses were re-classified as passive or active smokers if their urinary cotinine levels were >5 or >50 ng/mL, respectively. However, the smoking status of active and passive smokers was determined based on the self-reported questionnaire regardless of cotinine levels. Urinary cotinine, which is a primary metabolite of nicotine and has a half-life of 18–24 hours, is a reliable biomarker for smoking exposure. Measurement of urinary cotinine was conducted on the same day that the participants completed the survey and was performed via gas chromatographymass spectrometry (Perkin Elmer, Waltham, MA, USA) with a detection threshold of 0.28 ng/mL. The cutoff values of cotinine for active and passive smokers were >50 and 5 ng/mL, respectively, according to the values used in previous studies.

High-risk alcohol consumption was defined as ≥7 and ≥5 drinks on an occasion in men and women, respectively. Based on the frequency of highrisk alcohol consumption, we categorized alcohol consumption as <1 per week and ≥1 per week. Physical activity was assessed using the Global Physical Activity Questionnaire and participants who engaged in moderate activity for ≥150 min per week or in vigorous activity ≥75 min per week were defined as physically active, based on the WHO global recommendations for physical activity for health.

Physical examination (height, weight, and blood pressure) was performed by trained medical assistants in the specially-designed mobile center for KNHANES. The participants wore light clothing without shoes for measurement of height (m, SECA, Germany) and weight (kg, GL-6000-20, Korea). The body mass index was calculated as the weight divided by the square of height and was further categorized into three groups (<23, 23–24.9, and ≥25 kg/m^2^) based on the tailored criteria for the Korean population. Blood pressure was measured three times using a mercury sphygmomanometer (Baum, USA) and stethoscope (3M, USA) after the participant had rested for at least 5 min. Regardless of the value of the first measurement, the mean blood pressure of the last two measurements was calculated.

Venous blood samples were collected through venipuncture using a vacutainer needle and vacuum tubes. After serum separation, laboratory tests were performed to measure serum uric acid and serum creatinine levels. Uric acid levels were measured using colorimetry determination with the uricase–catalase system (Hitachi automatic analyzer 7600–210, Japan). The definition of hyperuricemia was serum urate level >7.0 mg/dL in men and >6.0 mg/dL in women. Kinetic Jaffe assays were used to measure serum creatinine levels (Hitachi autoanalyzer, model 7600, Tokyo, Japan). Based on serum creatinine levels, the glomerular filtration rate (GFR) was calculated using the Chronic Kidney Disease Epidemiology Collaboration equation. The definition of renal impairment was GFR <60 mL/min/1.73 m^2^.

### Statistical analysis

The sampling plan of KNAHNES followed a multistage clustered probability design to obtain a nationally representative sample of the Korean population. In addition, census data-based sample weights were constructed to address the complex survey design, non-responders, and post-stratification. Thus, complex survey design and sample weights were applied in all analyses.

The general characteristics of the participants were compared according to cotinine-verified smoking status (active, passive, and non-smokers). One-way analysis of variance was used for normally distributed continuous variables and chi-squared tests were used for categorical variables. Because urinary cotinine levels did not meet the assumption of normality, we used Kruskal–Wallis tests for comparisons. Mann–Whitney U tests were performed for pairwise comparisons of non-parametrically distributed variables. In addition, we compared smoking status based on WHO criteria with cotinine-verified smoking status by sex.

The associations between cotinine-verified smoking status and serum uric acid levels were estimated using a general linear regression model. We selected variables either with p<0.1 in the univariate analysis or variables significantly associated with hyperuricemia in previous studies as covariates for adjustment. Thus, Model 1 was adjusted for age; Model 2 was additionally adjusted for BMI, and GFR; Model 3 was additionally adjusted for area of residence, household income, education, blood pressure, alcohol consumption, and physical activity. To calculate p-values for trends, we used cotinine-verified smoking status as a continuous variable in the analysis model.

Multivariate logistic analysis was performed to evaluate the associations between cotinine-verified smoking status and hyperuricemia. The p-values for the trends were calculated to estimate the effects of smoking on hyperuricemia after adjusting for age, BMI, GFR, area of residence, household income, education, blood pressure, alcohol consumption, and physical activity.

Several sensitivity analyses were conducted. First, we divided active smokers into light and heavy tobacco exposure groups according to median urinary cotinine levels (1150 ng/mL in men and 670 ng/mL in women) and analyzed the dose-dependent association between tobacco exposure and hyperuricemia. Second, because the primary focus of this study was to examine the association between cotinine-verified smoking status and hyperuricemia, we ran the analysis without sample weights and clusters to determine whether the unweighted analysis altered the association observed in the analysis with sample weights. Third, another analysis was conducted to test for differences in the results when the smoking status was defined based on the WHO criteria. All tests were two-tailed and p<0.05 was considered statistically significant. All analyses were performed using IBM SPSS Statistics for Windows, version 24.0 (IBM Corp., Armonk, NY, USA).

## RESULTS

This study included 1171 cotinine-verified active smokers (22%; 38.7% of men and 8.8% of women) and 654 (12.3%; 11.9% of men and 12.6% of women) passive smokers. The overall active and passive smoking rates were 25.9 % (41.6% and 9.6% among men and women, respectively) and 12.9% (12.4% and 13.4% among men and women, respectively) in the weighted sample. The mean age of active smokers (43.1 years) was less than those in passive (47.5 years) and non-smokers (48.4 years). The active smoking rate was higher in men and most women were included in the non-smoking group (p<0.001). The education level of passive smokers was higher than those in active and non-smokers (p<0.001). Although active smokers had higher body mass index (p<0.001) and GFR (p=0.001) than non-smokers, these did not differ between active and passive smokers. Nonsmokers had lower diastolic blood pressure (p<0.001) and were less likely to drink alcohol heavily (p<0.001) than passive and active smokers. Significantly higher serum uric acid and urinary cotinine levels were observed in the active smoking group (p<0.001 for both). There were no differences in the area of residence, household income, systolic blood pressure, and physical activity between active, passive, and nonsmoking groups (Table 1).

**Table 1:** General characteristics of study participants according to cotinine-verified smoking status ^§^

*Variables*	*Overall (N=5329)*	*Non-smokers (N=3504)*	*Passive smokers (N=654)*	*Active smokers (N=1171)*	p*
**Age** (years), mean (SE)	46.8 (0.4)	48.4 (0.4)	47.5 (0.7)	43.1 (0.5)	<0.001^†‡^
**Sex,** % (SE)					<0.001*^†‡^
Men	50.8 (1.0)	38.1 (1.3)	48.9 (2.5)	81.7 (1.4)	
Women	49.2 (1.0)	61.9 (1.3)	51.1 (2.5)	18.3 (1.4)	
**Area of residence,** % (SE)					0.169
Urban	84.6 (1.8)	85.8 (1.8)	81.5 (2.8)	83.6 (2.5)	
Rural	15.4 (1.8)	14.2 (1.8)	18.5 (2.8)	16.4 (2.5)	
**Household income**					0.153
Low	36.3 (1.3)	36.5 (1.5)	36.0 (2.4)	35.6 (2.0)	
Middle	35.3 (1.0)	34.0 (1.2)	34.7 (2.1)	38.6 (1.9)	
High	28.4 (1.1)	29.5 (1.2)	29.3 (2.4)	25.8 (1.8)	
**Education,** % (SE)					<0.001*^†‡^
Middle school	39.6 (1.1)	40.9 (1.3)	32.4 (2.3)	40.1 (1.9)	
High school	36.1 (0.9)	32.7 (1.1)	41.9 (2.2)	41.4 (1.8)	
College or higher	24.3 (0.8)	26.4 (1.0)	25.7 (1.7)	18.5 (1.4)	
**Body mass index** (kg/m^2^), mean (SE)					0.001^†^
	24.0 (0.1)	23.8 (0.1)	24.2 (0.2)	24.3 (0.1)	
**Body mass index,** % (SE)					0.007^†^
<23.0	35.4 (0.9)	33.1 (1.1)	38.3 (2.3)	39.4 (1.8)	
23.0–24.9	22.8 (0.7)	23.5 (0.9)	20.2 (1.8)	22.7 (1.6)	
≥25.0	41.8 (0.9)	43.4 (1.1)	41.5 (2.3)	37.9 (1.7)	
**Systolic blood pressure,** mmHg	118.1 (0.3)	117.8 (0.4)	118.1 (0.7)	118.8 (0.5)	0.252
**Diastolic blood pressure,** mmHg	76.1 (0.2)	75.2 (0.2)	76.7 (0.5)	78.1 (0.3)	<0.001*^†^
**Alcohol consumption^¶^,** % (SE)					<0.001*^†‡^
<1 per week	77.7 (0.7)	86.7 (0.8)	77.2 (1.9)	56.3 (1.7)	
≥1 per week	22.3 (0.7)	13.3 (0.8)	22.8 (1.9)	43.7 (1.7)	
Physical activity**, % (SE)					0.637
Yes	48.6 (0.9)	48.5 (1.1)	50.6 (2.2)	48.0 (1.8)	
No	51.4 (0.9)	51.5 (1.1)	49.4 (2.2)	52.0 (1.8)	
**GFR,** mL/min/1.73m^2^	97.1 (0.4)	96.2 (0.4)	96.9 (0.8)	99.1 (0.6)	0.001^†^
**Uric acid,** mg/dL	5.11 (0.02)	4.91 (0.03)	5.05 (0.07)	5.62 (0.05)	<0.001^†‡^
**Urinary cotinine,** ng/mL					<0.001*^†‡^
Median value (IQR)	0.67 (0.34–42.00)	0.43 (0.26–0.69)	0.96 (0.51–3.46)	1080.00 (573.00–1630.00)	

Data are presented as weighted percentage (standard error [SE]) or weighted mean (SE) otherwise stated. GFR: glomerular filtration rate. IQR: interquartile range. P-values were calculated with the use of one-way analysis of variance for continuous variables, the chi-squared test for categorical variables and Kruskal-Wallis test for a non-parametrically distributed variable, respectively.

Values p<0.017 were considered significantly different between groups in pairwise comparisons using the Bonferroni correction: non-smokers vs passive smokers (*)

non-smokers vs active smokers (†)

and active smokers vs passive smokers (‡).

§ Smoking status was identified using self-reported data and urinary cotinine levels. High-risk alcohol consumption was defined as ≥7 drinks in men and ≥5 drinks in women on an occasion.

** Physical activity was defined as ≥150 min of moderate activity per day, or ≥75 min of vigorous activity per day based on the WHO recommendation.

With respect to discrepancy between self-report and cotinine-verified smoking status, while only 1.4% of cotinine-verified male active smokers answered never smokers in self-reported questionnaires, 31.8% of female active smokers were never smokers based on self-report (Table 2).

**Table 2:** Comparison of cotinine-verified smoking status with self-reported smoking status based on WHO

*Smoking status*	*Self-reported*	*Cotinine-verified*		
	*n (%)*	*Non-smokers n (%)*	*Passive smokers n (%)*	*Active smokers n (%)*
**Men**				
Never smoker	608 (25.9)	478 (41.2)	117 (41.8)	13 (1.4)
Past smoker	906 (38.6)	681 (58.8)	163 (58.2)	62 (6.8)
Current smoker	835 (35.5)	0 (0)	0 (0)	835 (91.8)
Total	2349 (100)	1159 (100)	280 (100)	910 (100)
**Women**				
Never smoker	2699 (90.6)	2263 (96.5)	353 (94.4)	83 (31.8)
Past smoker	125 (4.2)	82 (3.5)	21 (5.6)	22 (8.4)
Current smoker	156 (5.2)	0 (0)	0 (0)	156 (59.8)
Total	2980 (100)	2345 (100)	374 (100)	261 (100)

**Figure 1:**
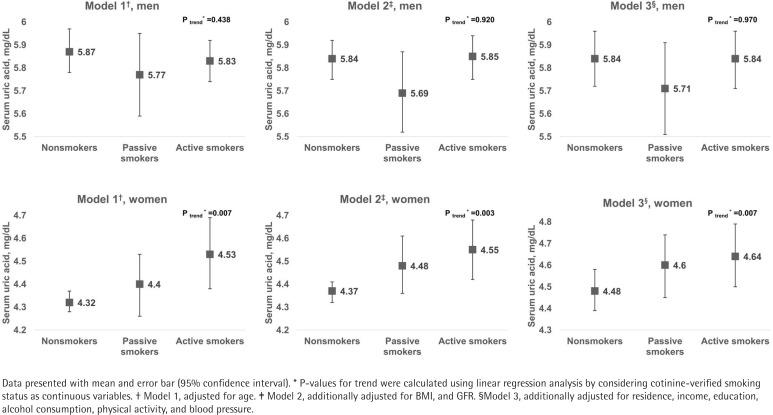
Serum uric acid by smoking status and gender, for Models 1 to 3

Serum uric acid concentrations according to cotinine-verified smoking status are shown in Figure 1. Higher uric acid levels were observed with increasing tobacco exposure in women (p-trend=0.007), but not in men. This incremental trend in uric acid level remained after adjusting for BMI, and GFR (p-trend=0.003), and further adjusting for area of residence, household income, education, alcohol consumption, physical activity, and blood pressure in the multivariate model (p-trend=0.007).

The prevalence of hyperuricemia was 9.4% in nonsmokers, 12.1% in passive smokers, and 15.6% in active smokers (Table 3). Female active smokers were more likely to have hyperuricemia than non-smokers while there was no difference in hyperuricemia rates among men. In the age-adjusted model, the risk of hyperuricemia tended to increase with greater tobacco exposure only in women (p-trend=0.004). Although the odds ratio for hyperuricemia increased slightly among female passive smokers, the strength of the association was slightly attenuated among active smokers in Models 2 (p-trend=0.006) and 3 (p-trend=0.016).

**Table 3:** Prevalence of hyperuricemia and association between cotinine-verified smoking status and hyperuricemia

	*Non-smokers*	*Passive smokers*	*Active smokers*	*p-trend*
Men				
Prevalence of hyperuricemia, %	16.7 (14.2–19.4)	17.8 (13.4–23.3)	17.2 (14.7–19.9)	0.133*
Model 1^†^	Ref.	1.08 (0.73–1.58)	0.97 (0.74–1.26)	0.808**
Model 2^‡^	Ref.	1.02 (0.68–1.53)	1.04 (0.78–1.39)	0.790**
Model 3^§^	Ref.	1.04 (0.66–1.52)	1.00 (0.74–1.35)	0.995**
**Women**				
Prevalence of hyperuricemia, %	4.9 (4.0–6.1)	6.6 (4.2–10.4)	8.4 (5.3–13.0)	0.007*
Model 1^†^	Ref.	1.45 (0.86–2.44)	2.00 (1.19–3.39)	0.004**
Model 2^‡^	Ref.	1.72 (0.99–2.98)	1.89 (1.07–3.34)	0.006**
Model 3^§^	Ref.	1.61 (0.90–2.86)	1.81 (1.03–3.19)	0.016**

Data are presented as percentage or odds ratio (95% confidence interval). * P-values for trend were calculated using linear-by-linear test.

** P-values for trend were calculated using logistic regression analysis by considering cotinine-verified smoking status as continuous variables.

† Model 1 adjusted for age;

‡ Model 2 additionally adjusted for BMI, and GFR;

§ Model 3 additionally adjusted for residence, income, education, alcohol consumption, physical activity, and blood pressure.

When we divided active smokers into light and heavy tobacco exposure groups based on median cotinine levels, more robust dose-dependent association between smoking exposure and risk of hyperuricemia was observed among women, but not among men (Supplementary file, Table S1). Furthermore, analysis of the unweighted sample showed a slightly increased magnitude of the association among women; however, the incremental trend between smoking exposure and the risk of hyperuricemia did not change (Supplementary file, Table S2). In the analysis using self-reported smoking status based on WHO classification to test the association between smoking and hyperuricemia revealed that active smoking was significantly associated with hyperuricemia among women and no significant associations were observed among men (Supplementary file, Table S3).

## DISCUSSION

To our knowledge, this is the first study to evaluate the association between cotinine-verified smoking status and serum uric acid level and hyperuricemia in a nationally representative sample. We found that cotinine-verified active and passive smoking were positively associated with serum uric acid levels only in women. In addition, we observed a dose-response relationship between cotinine-verified smoking exposure and the risk of hyperuricemia only in women. However, tobacco exposure did not have a significant effect on serum uric acid concentration or risk of hyperuricemia in men.

Assessing smoking status based on self-reporting is unreliable due to substantial discrepancies between self-reports and biochemically verified smoking status, especially in women. In line with our study results, several studies conducted in Korea showed that female smokers tended to under-report their smoking status in self-reported questionnaires^17,18^. The passive smoking rate also tended to be underestimated in self-reports^19^. Therefore, smoking rates calculated solely based on self-reporting data are likely to underestimate the number of active and passive smokers. To address this misclassification issue, we confirmed smoking status with the use of urinary cotinine, which is an objective biochemical marker of smoking exposure. However, urinary cotinine can only reflect smoking exposure within the previous 48–72 hours because of the short half-life of cotinine; thus, participants who had been exposed to tobacco more than three days before the test might have been misclassified as non-smokers rather than active or passive smokers. Therefore, we initially determined the smoking status of the participants based on their self-reports and verified their status using urinary cotinine levels.

Consistent with the results of previous studies, the current study found that cotinine-verified active and passive smoking were independently associated with increasing serum uric acid level and risk of hyperuricemia only in women. An analysis of the National Health and Nutrition Examination Survey (NAHNES) III cohort showed that smoking was positively correlated with uric acid concentration only in women after adjusting for multiple covariates (age, alcohol, BMI, blood pressure, and ethnicity)^20^. Another report from the Coronary Artery Risk Development in Young Adults (CARDIA) study showed that women who were smoking at baseline had a significantly higher risk for increased uric acid levels over 10 years than women who were non-smokers; however, this finding was not observed among men^13^. Comparable findings were also reported in a study conducted in Japan showing increased serum uric acid among the smoking population^14^. Although the reason for the positive association between smoking exposure and hyperuricemia only in women is unclear, prominent renal function declining in women with IgA nephritis might, at least partly, be related to this finding^21^. In addition, metabolic impairment due to smoking exposure could play a significant role in the risk for hyperuricemia, which is different between women and men^22^.

However, several other studies on the influence of smoking on uric acid levels reported the opposite results. A decreased risk of hyperuricemia was observed among smokers regardless of sex in the Framingham Heart Study^15^ and the protective effects of smoking on serum uric acid and hyperuricemia were reported in the Tromsø^23^ and Tunisian^24^ studies. Another study from Japan also showed that cigarette smoking was significantly associated with decreasing serum uric acid and risk of hyperuricemia10, a finding also reported in a study from China^25^. However, a few previous studies failed to show a significant association between tobacco exposure and serum uric acid concentrations^26,27^.

Active and passive smoking were positively associated with the risk for hyperuricemia compared to non-smoking and the magnitude of the risk increased with increased smoking exposure. Previous studies on the effects of smoking on hyperuricemia reported conflicting findings. A Chinese study showed that current smokers had a 1.19-fold increased risk for hyperuricemia, while an analysis of the Atherosclerosis Risk in Communities (ARIC) cohort study reported that the likelihood of hyperuricemia was 1.27 times higher among the smoking population than that in non-smokers^28^. However, in a Japanese cohort study, male smokers showed a 35% lower risk of hyperuricemia than male non-smokers^16^. Moreover, several other studies observed no significant association between smoking and hyperuricemia.

Although the reasons for the conflicting finding on the effect of smoking on serum uric acid level and hyperuricemia are unclear, inconsistent definitions of smoking exposure might be related to this discrepancy. For example, smoking status categorized as current smoking and non-smoking would likely include most passive smokers in the non-smoking group, which would decrease the magnitude of the effect of smoking on serum uric acid level and hyperuricemia. Misclassification of smoking status, particularly in women, based on self-reporting may also contribute to the inconsistency in study results. Considering that half of female active smokers did not reveal their smoking status in self-reporting, misclassification of active smokers as non-smokers might decrease the strength of the association between smoking and uric acid levels, resulting in a lack of significant difference between active smoking and non-smoking groups. Moreover, the time from smoking exposure to uric acid measurement also contributes to conflicting findings. One study reported decreased serum uric acid levels after 5 min of smoking, which returned to baseline levels after 60 min^29^. Although this finding suggests that the time of the last smoking exposure should be considered when evaluating the effects of smoking on uric acid levels, most previous studies did not consider its potentially significant impact. However, these theories do not fully explain the protective effects of smoking against increased uric acid concentration and hyperuricemia observed in previous studies. Further experimental and clinical studies are warranted to address these discrepant findings.

Several mechanisms have been proposed to explain the negative impact of smoking on serum uric acid levels and hyperuricemia. First, renal damage caused by chronic exposure to tobacco smoking could contribute to increased serum uric acid levels. Smoking exposure induced mesangial cell proliferation and increased production of fibronectin, TGF-β1, which play an important role in the progression of chronic kidney disease^30,31^. Degenerative changes in the proximal tubules and mesangial proliferation were observed in rats exposed to tobacco smoking for more than 2 months; these rats also exhibited significantly elevated serum uric acid levels compared to the nonexposed rats^32^. This finding suggests that renal damage caused by smoking exposure might be associated with hyperuricemia. Furthermore, the negative effects on renal function due to exposure to cadmium and lead in cigarette smoke could impair renal function, leading to increased uric acid concentrations^33^. Finally, physiologic response to oxidative stress from cigarette smoking could also explain the negative influence of smoking on serum uric acid levels. Because uric acid has anti-oxidative effects, oxidative stress caused by reactive oxygen species from tobacco smoking components results in increased uric acid production to counteract these oxidative agents^34^.

### Limitations

The present study has several limitations. First, because the cross-sectional design could not address temporality, caution is necessary when interpreting the causal relationship of the findings. However, it is more plausible that cigarette smoking affects serum uric acid levels rather than vice versa. Second, although we verified self-reported smoking status using urinary cotinine levels, a small portion of the study population might have been misclassified because there is no consensus on the cutoff values for urinary cotinine levels in active, passive, and nonsmokers. Nevertheless, the cut-off values (≥50 and ≥5 ng/mL) used in the present study were widely accepted in previous epidemiologic studies of the Korean population and other studies support these values for the categorization of smoking status^17,18^. Third, because we could not determine the use of uric acid-lowering agents such as allopurinol, febuxostat, or rasburicase, the number of participants with hyperuricemia might be underestimated. However, it is unlikely that the use of these medications significantly affected the study results considering the prevalence of gout in Korea (0.76%). Fourth, due to the low response rate to the frequent food intake questionnaire, we could not include dietary factors such as the frequency of high-purine diet intake, seafood intake etc., which might be potential cofounders of the analysis model.

## CONCLUSIONS

Cotinine-verified active and passive smoking were significantly associated with serum uric acid levels in women and the risk of hyperuricemia increased in a dose-response manner with increasing smoking exposure. This study might provide evidence to support the importance of smoking cessation in women with gout and further studies are necessary to elucidate the underlying mechanism of the observed association between smoking and serum uric acid levels.
