# Supplementary material for: Association of urinary cotinine-verified smoking status with hyperuricemia: Analysis of population-based nationally representative data
Source: Tob Induc Dis. 2020 Oct 6;18:84. doi: 10.18332/tid/127269 (PMC7557215; doi:10.18332/tid/127269)
Supplement: Supplementary file 1 [file TID-18-84-s1.pdf]

STROBE Statement—Checklist of items that should be included in reports of ***cross-sectional studies***

	Item No	Recommendation
**Title and abstract**	1	(*a*) Indicate the study's design with a commonly used term in the title or the abstract : **checked, page 3**
		(*b*) Provide in the abstract an informative and balanced summary of what was done and what was found : **checked, page 3**
**Introduction**		
Background/rationale	2	Explain the scientific background and rationale for the investigation being reported : **checked, page 4**
Objectives	3	State specific objectives, including any prespecified hypotheses : **checked, page 4**
**Methods**		
Study design	4	Present key elements of study design early in the paper : **checked, Page 6**
Setting	5	Describe the setting, locations, and relevant dates, including periods of recruitment, exposure, follow-up, and data collection : **checked, Page 6**
Participants	6	(*a*) Give the eligibility criteria, and the sources and methods of selection of participants : **checked, Page 6**
Variables	7	Clearly define all outcomes, exposures, predictors, potential confounders, and effect modifiers. Give diagnostic criteria, if applicable : **checked, page 7-8**
Data sources/ measurement	8*	For each variable of interest, give sources of data and details of methods of assessment (measurement). Describe comparability of assessment methods if there is more than one group : **checked, page 7-8**
Bias	9	Describe any efforts to address potential sources of bias : **checked, page 11**

Study size	10	Explain how the study size was arrived at : **N/A**
Quantitative variables	11	Explain how quantitative variables were handled in the analyses. If applicable, describe which groupings were chosen and why : **checked, page 7-8**
Statistical methods	12	(*a*) Describe all statistical methods, including those used to control for confounding : **checked, page 9**
		(*b*) Describe any methods used to examine subgroups and interactions : **checked, page 9**
		(*c*) Explain how missing data were addressed : **checked, page 7-8**
		(*d*) If applicable, describe analytical methods taking account of sampling strategy, **N/A**
		(*e*) Describe any sensitivity analyses : **N/A**
**Results**		
Participants	13*	(a) Report numbers of individuals at each stage of study—eg numbers potentially eligible, examined for eligibility, confirmed eligible, included in the study, completing follow-up, and analysed : **Page 11**
		(b) Give reasons for non-participation at each stage : **checked, page 11**
		(c) Consider use of a flow diagram : **N/A**
Descriptive data	14*	(a) Give characteristics of study participants (eg demographic, clinical, social) and information on exposures and potential confounders : **checked, page 11**
		(b) Indicate number of participants with missing data for each variable of interest : **checked, Page 6**

Outcome data	15*	Report numbers of outcome events or summary measures : **checked, page 11-12**
Main results	16	(*a*) Give unadjusted estimates and, if applicable, confounder-adjusted estimates and their precision (eg, 95% confidence interval). Make clear which confounders were adjusted for and why they were included : **checked, page 11-12**
		(*b*) Report category boundaries when continuous variables were categorized : **checked, page 7-8**
		(*c*) If relevant, consider translating estimates of relative risk into absolute risk for a meaningful time period : **N/A**
Other analyses	17	Report other analyses done—eg analyses of subgroups and interactions, and sensitivity analyses : **checked, page 10**
**Discussion**		
Key results	18	Summarise key results with reference to study objectives : **checked, page 13-14**
Limitations	19	Discuss limitations of the study, taking into account sources of potential bias or imprecision. Discuss both direction and magnitude of any potential bias : **checked, page 17**
Interpretation	20	Give a cautious overall interpretation of results considering objectives, limitations, multiplicity of analyses, results from similar studies, and other relevant evidence : **checked, page 13-15**
Generalisability	21	Discuss the generalisability (external validity) of the study results : **checked, page17**
**Other information**		

Funding	22	Give the source of funding and the role of the funders for the present study and, if applicable, for the original study on which the present article is based : **checked page 1**

*Give information separately for exposed and unexposed groups.

Note: An Explanation and Elaboration article discusses each checklist item and gives methodological background and published examples of transparent reporting. The STROBE checklist is best used in conjunction with this article (freely available on the Web sites of PLoS Medicine at http://www.plosmedicine.org/, Annals of Internal Medicine at http://www.annals.org/, and Epidemiology at http://www.epidem.com/). Information on the STROBE Initiative is available at www.strobe-statement.org.

Supplementary Table S1. Prevalence of hyperuricemia and association between cotinine-verified smoking status and hyperuricemia after dividing active smokers into light-smokers and heavy-smokers based on the median urine cotinine level (1150ng/ml in men and 670ng/ml in women respectively)

	Nonsmokers	Past smokers	Light active smokers	Heavy active smokers	P for trend
Men					
Prevalence of hyperuricemia, %	16.7(14.2,19.4)	17.8(13.4,23.3)	21.6(16.3,27.9)	15.7(13.0,18.8)	0.370
Model 1†	Reference	1.08(0.73,1.58)	1.28(0.86,1.91)	0.87(0.65,1.16)	0.486
Model 2‡	Reference	1.02(0.68,1.53)	1.22(0.80,1.87)	0.97(0.71,1.33)	0.969
Model 3§	Reference	1.04(0.66,1.52)	1.20(0.77,1.87)	0.93(0.78,1.28)	0.803
Women					
Prevalence of hyperuricemia, %	4.9(4.0,6.1)	6.6(4.2,10.4)	9.6(5.6,16.0)	7.2(3.8,13.3)	0.010
Model 1†	Reference	1.45(0.86,2.44)	2.25(1.19,4.23)	1.75(0.84,3.61)	0.006
Model 2‡	Reference	1.72(0.99,2.98)	1.68(0.75,3.75)	2.21(1.12,4.37)	0.004
Model 3§	Reference	1.61(0.90,2.86)	1.60(0.74,3.48)	2.13(1.03,4.41)	0.011

Data were presented with percentage or odds ratio (95% confidence interval)

* P for trend were calculated using linear regression analysis by considering cotinine-verified smoking status as continuous variables

† Model 1 adjusted for age, ‡ Model 2 additionally adjusted for BMI, and GFR § Model 3 additionally adjusted for residence, income, education, alcohol consumption, physical activity, and blood pressure

Supplementary Table S2. Prevalence of hyperuricemia and association between cotinine-verified smoking status and hyperuricemia with unweighted sample

	Nonsmokers	Past smokers	Active smokers	P for trend
Men				
Prevalence of hyperuricemia, %	15.4(13.5,17.6)	16.8(12.9,21.6)	17.9(15.6,20.5)	0.133
Model 1†	Reference	1.05(0.74,1.50)	1.09(0.86,1.38)	0.491
Model 2‡	Reference	0.98(0.68,1.42)	1.20(0.94,1.54)	0.156
Model 3§	Reference	0.94(0.65,1.37)	1.14(0.88,1.47)	0.341
Women				
Prevalence of hyperuricemia, %	5.3(4.4,6.3)	6.1(4.1,9.1)	9.6(6.6,13.8)	0.007
Model 1†	Reference	1.24(0.78,1.97)	2.22(1.40,3.51)	0.001
Model 2‡	Reference	1.42(0.87,2.30)	2.18(1.32,3.61)	0.002
Model 3§	Reference	1.30(0.79,2.14)	1.93(1.15,3.26)	0.012

Data were presented with percentage or odds ratio (95% confidence interval)

* P for trend were calculated using linear regression analysis by considering cotinine-verified smoking status as continuous variables

† Model 1 adjusted for age, ‡ Model 2 additionally adjusted for BMI, and GFR § Model 3 additionally adjusted for residence, income, education, alcohol consumption, physical activity, and blood pressure

Supplementary Table S3. Prevalence of hyperuricemia and association between self-reported smoking status and hyperuricemia

	Nonsmokers	Past smokers	Active smokers	P for trend
Men				
Prevalence of hyperuricemia, %	17.5(14.2,21.3)	16.4(13.6,19.6)	17.2(14.6,20.2)	0.189
Model 1[†]	Reference	1.11(0.79,1.54)	1.01(0.75,1.37)	0.988
Model 2[‡]	Reference	1.06(0.75,1.51)	1.13(0.82,1.57)	0.452
Model 3[§]	Reference	1.06(0.74,1.53)	1.09(0.78,1.53)	0.635
Women				
Prevalence of hyperuricemia, %	5.2(4.2,6.4)	4.5(2.0,9.5)	10.6(6.3,17.4)	0.001
Model 1[†]	Reference	1.02(0.43,2.39)	2.57(1.39,4.76)	0.004
Model 2[‡]	Reference	0.95(0.38,2.37)	2.66(1.45,4.89)	0.004
Model 3[§]	Reference	0.94(0.36,2.46)	2.62(1.43,4.78)	0.004

Data were presented with percentage or odds ratio (95% confidence interval)

* P for trend were calculated using linear regression analysis by considering cotinine-verified smoking status as continuous variables

† Model 1 adjusted for age, ‡ Model 2 additionally adjusted for BMI, and GFR § Model 3 additionally adjusted for residence, income, education, alcohol consumption, physical activity, and blood pressure

© 2020 Kim Y. and Kang J.
